# Universal newborn hearing screening in the Lazio region, Italy

**DOI:** 10.1186/s13052-018-0534-5

**Published:** 2018-08-24

**Authors:** Rosaria Turchetta, Guido Conti, Pasquale Marsella, Maria Patrizia Orlando, Pasqualina Maria Picciotti, Simonetta Frezza, Francesca Yoshie Russo, Alessandro Scorpecci, Maria Gloria Cammeresi, Sara Giannantonio, Antonio Greco, Massimo Ralli

**Affiliations:** 1 Department of Sense Organs, Sapienza University of Rome, Rome, Italy; 2 Department of Head and Neck Surgery, Institute of Otorhinolaryngology, Catholic University of Sacred Heart, Rome, Italy; 3 Department of Surgery, Audiology and Otosurgery Unit, Bambino Gesù Pediatric Hospital, Rome, Italy; 4 Department of Pediatrics, Division of Neonatology, Catholic University of Sacred Heart, Rome, Italy

**Keywords:** Universal newborn hearing screening, Hearing loss, A-TEOAE, ABR

## Abstract

**Background:**

The introduction of Universal Newborn Hearing Screening (UNHS) programs has drastically contributed to the early diagnosis of hearing loss in children, allowing prompt intervention with significant results on speech and language development in affected children. UNHS in the Lazio region has been initially deliberated in 2012; however, the program has been performed on a universal basis only from 2015. The aim of this retrospective study is to present and discuss the preliminary results of the UNHS program in the Lazio region for the year 2016, highlighting the strengths and weaknesses of the program.

**Methods:**

Data from screening facilities in the Lazio region for year 2016 were retrospectively analyzed. Data for Level I centers were supplied by the Lazio regional offices; data for Level II and III centers were provided by units that participated to the study.

**Results:**

During 2016, a total of 44,805 babies were born in the Lazio region. First stage screening was performed on 41,821 children in 37 different birth centers, with a coverage rate of 93.3%. Of these, 38.977 (93.2%) obtained a “pass” response; children with a “refer” result in at least one ear were 2844 (6.8%). Data from Level II facilities are incomplete due to missing reporting, one of the key issues in Lazio UNHS. Third stage evaluation was performed on 365 children in the three level III centers of the region, allowing identification of 70 children with unilateral (40%) or bilateral (60%) hearing loss, with a prevalence of 1.6/1000.

**Conclusions:**

The analysis of 2016 UNHS in the Lazio region allowed identification of several strengths and weaknesses of the initial phase of the program. The strengths include a correct spread and monitoring of UNHS among Level I facilities, with an adequate coverage rate, and the proper execution of audiological monitoring and diagnosis among Level III facilities. Weakness, instead, mainly consisted in lack of an efficient and automated central process for collecting, monitoring and reporting of data and information.

## Background

Hearing loss is one of the most common defects at birth occurring in about 1.4 babies per 1000 newborns [1, 2]. Hearing impairment can affect a child’s ability to develop speech, language and social skills [3]; a late diagnosis can severely impact on the future life of the child with serious disability and related high costs [4–6]. A recent study in the Italian population estimated that the lifetime mean cost for a subject with profound pre-lingual deafness is around €700.000 [7].

The early diagnosis and intervention in children with hearing loss at < 6 months of age leads to significantly better outcomes for speech and language development compared to non-treated children [8, 9]. Early diagnosis is based on Universal Newborn Hearing Screening (UNHS) programs; intervention relies on the use of hearing aids and cochlear implants [10]. It has been largely demonstrated that UNHS is the only effective way to evaluate the largest portion of population; when neonatal hearing screening is restricted to high risk groups, hearing impairment remains undiagnosed in 30–50% of infants [10–15]. Furthermore, in the absence of a UNHS program, moderate to severe hearing loss is not identified before a mean age of two years and not treated earlier than 40 months, while mild hearing impairment is identified even later, sometimes at school age [16].

**Fig. 1:**
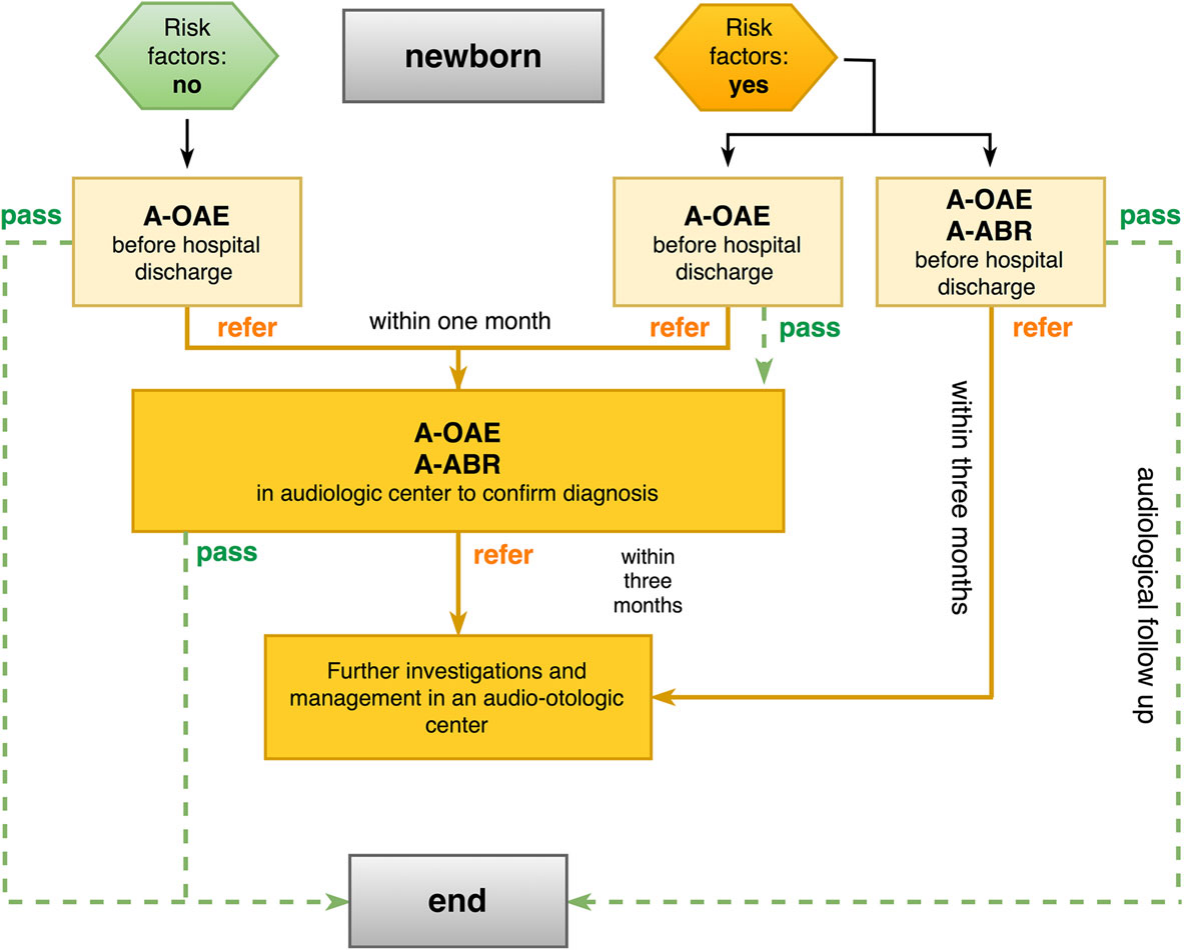
Graphical representation of the recommended algorithm for Universal Newborn Hearing Screening in the Lazio region

Several Italian regions have introduced UNHS during the last decade, with different results and methodologies. In 2017, the Italian Ministry of Health introduced UNHS among the Essential Levels of Assistance (ELA). Lazio is a region in central Italy with an area of about 18,000 km^2^, a population of about six million inhabitants and 50.000 newborns every year. In the Lazio region, UNHS has been introduced in 2012 with Regional Council Resolution n. 115/2012 “Linea d'azione screening uditivo neonatale universale. Programma di attivazione e messa a regime”, a document based on the national and international guidelines [14, 17]. However, UNHS has been performed regularly on a universal basis only from 2015. A graphical representation of the recommended algorithm for Universal Newborn Hearing Screening in the Lazio region is shown in Fig. 1.

The aim of this retrospective study is to present and discuss the results of the 2016 UNHS program in the Lazio region, highlighting the main strengths and weaknesses of the initial phase of the program.

## Methods

### Operative and geographical organization

UNHS was executed in all birth centers and neonatal intensive care units (NICU) of the region, homogenously distributed in the territory. Screening facilities, defined by the regional government in 2012 according to international guidelines, were divided into three different levels:

- *Level I*: includes most of birth centers and NICU, that can execute Transient Evoked Otoacoustic Emissions (TEOAE) only. TEOAE testing can be executed by trained nursing personnel, neonatologists and audiometrists. In the Lazio region, there are 37 Level I facilities. The original regional plan included 44 Level I centers; however, seven smaller centers were subsequently merged.
- *Level II*: these facilities, mainly available in middle and large hospitals, execute TEOAE and Automated Auditory Brainstem Responses (A-ABR). Tests can be executed by specifically trained personnel including pediatric nurses, audiometrists, pediatricians, audiologists, otolaryngologists. In the Lazio region, there are 11 Level II facilities.
- *Level III*: Level III centers, also named “reference centers”, can perform the whole clinical and audiological evaluation in children, including testing clinical TEOAE, Distortion Product Otoacoustic Emissions (DPOAE) and ABR, and lead to a complete diagnosis of hearing loss and the activation of the habilitation. In the Lazio region there are three Level III centers: the Policlinico Umberto I Hospital, the Agostino Gemelli Hospital, and the Bambino Gesù Pediatric Hospital.

Detailed data on facilities included in the UNHS in the Lazio region, and the corresponding Level III center are outlined in Table 1.

**Table 1:** Organization of universal newborn hearing screening in the Lazio region

Territory	Level I	Level II	Level III
Rome	San Giovanni Calibita – FBF	San Giovanni Calibita – FBF	Bambino Gesù Hospital
Rome	Quisisana		
Rome	Villa Mafalda		
Rome	Villa Margherita		
Rome	Mater Dei		
Rome	Sant’Eugenio Hospital	Sant’Eugenio Hospital	Policlinico Umberto I Hospital
Rome	San Giovanni Hospital		
Rome	Vannini Hospital		
Rome	Fabia Mater		
Rome	Annunziatella		
Rome	Policlinico Umberto I	Policlinico Umberto I Hospital	
Rome	Policlinico Casilino		
Rome	Pertini Hospital		
Colleferro	Parodi Delfino		
Palestrina	Coniugi Berardini		
Tivoli	San Giovanni Evangelista		
Rome	Grassi Hospital	Grassi Hospital	
Rome	Villa Pia		
Rome	Città di Roma		
Rome	San Camillo Hospital	San Camillo Hospital	
Rome	Policlinico Gemelli	Policlinico Gemelli	Policlinico Gemelli
Rome	Santo Spirito Hospital		
Rome	Santa Famiglia		
Rome	Cristo Re		
Rome	San Pietro - FBF	Bambino Gesù Hospital	Bambino Gesù Hospital
Rome	Aurelia Hospital		
Rome	Santa Maria di Leuca		
Rome	San Filippo Neri		
Civitavecchia	San Paolo Hospital		
Anzio	P.O. Anzio Nettuno		
Albano Laziale	P.O. Albano		
Marino	San Giuseppe		
Velletri	Civile Paolo Colombo		
Latina	P.O. Latina Nord	P.O. Latina Nord	
Terracina	P.O. Latina Centro		
Formia	P.O. Latina Sud		
Aprilia	Città di Aprilia		
Tarquinia	Civile di Tarquinia	Belcolle Hospital	Policlinico Gemelli
Viterbo	Belcolle Hospital		
Rieti	P.O. Rieti	P.O. Rieti	
Frosinone	Spaziani Hospital	Spaziani Hospital	
Alatri	San Benedetto		
Sora	Santissima Trinità		
Cassino	Santa Scolastica		

Names and distribution of Level I, II and III facilities in the Lazio region

### Screening protocol

UNHS should be performed in all newborns within 72 h after birth before discharge. Screening operative protocol is divided into three stages.

1. *First Stage*: initial screening performed on all newborns in both ears using TEOAE by trained personnel. TEOAE testing should be performed in a quiet environment when babies are asleep or feeding. In the case of a bilateral “pass” response, the screening is considered completed. In the case of a “refer” response in one or both ears, it is recommended to repeat the test before discharge. Newborns with a “refer” at this first stage evaluation are addressed to second stage evaluation within 15 days.
2. *Second Stage*: includes children that do not pass first stage screening in at least one ear and babies with a family history of sensorineural hearing loss or pregnancy or auditory neuropathy risk factors, according to the Joint Committee on Infant Hearing (JCIH) 2007, and is executed using A-ABR. Intrauterine risk factors include maternal infections, such as Toxoplasmosis, Syphilis, HIV, Hepatitis B, Rubella, Citomegalovirus, Herpes Simplex during pregnancy or delivery; admission to a NICU greater than five days; prematurity (< 37 weeks); exposure to ototoxic medications such as gentamycin, tobramycin and furosemide; hyperbilirubinemia that requires transfusion; syndromes associated with hearing impairment such as Pendred, Usher, Waardenburg, Neurofibromatosis; and craniofacial anomalies, including those involving the pinna, ear canal, ear tags, ear pits, and temporal bone anomalies (Table 2). All babies must be tested within the first month of life. In case of a bilateral “pass” response at A-ABR, the screening is considered completed. However, it is recommended that babies with known risk factors are monitored with TEOAE, ABR, acoustic immittance testing at regular intervals every six months for the first three years, then every twelve months for the following three years to identify children with late or progressive hearing loss.
3. *Third Stage (clinical audiological evaluation)*: includes children that fail second stage testing in one or both ears. It is executed by level III facilities by means of case history, otoscopy, clinical TEOAE and/or DPOAE, ABR recording and impedance under supervision of experienced otolaryngologists or audiologists. All babies must be tested within the third month of life and should begin a prosthetic and rehabilitation treatment within six months of age.

**Table 2:** Audiological risk factors

Audiological risk factor *—* JCIH	
Family history of hereditary childhood sensorineural hearing loss	
In-utero infection (e.g., rubella, cytomegalovirus, syphilis, toxoplasmosis, herpes) Craniofacial anomalies	
Low birth weight (b1500 g or 3.3 lb)	
Hyperbilirubinemia at levels requiring exchange transfusion	
Bacterial meningitis	
Exposure to ototoxic medications	
Mechanical ventilation lasting 5 days or longer	
Stigmata or other findings associated with a syndrome known to include a sensorineural and/or conductive hearing loss	
Apgar scores of 0–4 at 1 min or 0–6 at 5 min	
Admission to the Neonatal Intensive Care Unit (NICU)	
Parent/caregiver concern regarding hearing, speech, language, and/or developmental delay	
Head trauma associated with loss of consciousness or skull fracture	
Recurrent or persistent otitis media with effusion lasting for at least 3 months	

Audiological risk factor according to the Joint Committee on Infant Hearing (JCIH)

### Data collection

The following data for each Level I facility were provided by regional offices: number of children born alive; number of children tested; number of children with a “pass” response; number of children with a “refer” response. Such data were digitally tracked and transmitted by Level I facilities to the monitoring offices of the Lazio region.

Data from Level II facilities were provided only by four centers: Policlinico Umberto I, Policlinico Gemelli, Ospedale San Camillo Forlanini, Ospedale Pediatrico Bambino Gesù upon request. Data included: number of children referred from Level I facilities, although the referring center was not specified; number of children tested; number of children with a “pass” response; number of children with a “refer” response.

Complete data were provided from individual registries of the three Level III centers of the Lazio region: Policlinico Umberto I, Policlinico Gemelli, Ospedale Pediatrico Bambino Gesù. Data included: number of children referred to Level III centers; number of children tested; number of children with unilateral/bilateral hearing loss; degree of hearing loss (mild/moderate; severe/profound). No information was provided on risk factors of babies included in the screening protocol.

## Results

During 2016, a total of 44,805 babies were born in the Lazio region. First stage screening was performed on 41,821 in 37 different birth centers. They included 26 public hospitals and 11 private hospitals and clinics. Overall coverage rate was 93.3%. Territory distribution was as follows: 25 birth centers were in central Lazio (67.6%); eight in southern Lazio (21.6%) and four in northern Lazio (10.8%). Among birth centers in central Lazio, 21 (84%) were in the city of Rome. When first stage screening was not possible in a birth center, children were referred to other level I or II centers. Children that obtained a “pass” response at first stage screening were 38,977 (93.2%); children with a “refer” result in at least one ear were 2844 (6.8%). Detailed data on first stage screening performed during 2016 in the Lazio region are shown in Table 3.

**Table 3:** First stage screening results

BIRTH CENTERS	BORN ALIVE	TESTED	% TESTED	PASS	REFER	% REFER
San Giovanni FBF	3643	3580	98,27%	3552	28	0,78%
Quisisana	84	41	48,81%	41	0	0,00%
Villa Mafalda	30	5	16,67%	–	–	–
Villa Margherita	132	94	71,21%	94	0	0,00%
San Giovanni Addolorata	337	337	100,00%	289	48	14,24%
Sant’Eugenio	1107	1102	99,55%	1018	84	7,62%
M.G. Vannini	300	291	97,00%	279	12	4,12%
Fabia Mater	1730	622	35,95%	600	22	3,54%
Pol. Umberto I	1641	1551	94,52%	1307	244	15,73%
Pol. Casilino	2746	2710	98,69%	2613	97	3,58%
Sandro Pertini	1152	1075	93,32%	1045	30	2,79%
Con. Bernardini (Palestrina)	705	697	98,87%	670	27	3,87%
San Giovanni E. (Tivoli)	678	666	98,23%	605	61	9,16%
GB. Grassi	1711	1700	99,36%	1680	20	1,18%
Città di Roma	1278	1269	99,30%	1264	5	0,39%
San Camillo Forlanini	2580	2645	102,52%	2352	293	11,08%
S. Spirito	643	633	98,44%	622	11	1,74%
Santa Famiglia	1833	1829	99,78%	1815	14	0,77%
Cristo Re	1929	1316	68,22%	1272	44	3,34%
Policlinico Gemelli	3697	3697	100,00%	3323	374	10,12%
San Filippo Neri	951	933	98,11%	820	113	12,11%
Ospedale San Pietro FBF	4545	3954	87,00%	3954	604	15,28%
Aurelia Hospital	444	424	95,50%	414	10	2,36%
S Maria di Leuca	94	85	90,43%	85	0	0,00%
O. S. Paolo	427	427	100,00%	425	2	0,47%
P.O. Anzio Nettuno	545	532	97,61%	527	5	0,94%
P.O. Albano Genzano	850	809	95,18%	809	0	0,00%
P.O. Paolo Colombo	530	516	97,36%	512	4	0,78%
P.O. Latina Nord	1705	1911	112,08%	1909	1	0,05%
P.O. Latina Centro	836	836	100,00%	835	1	0,12%
P.O. Latina Sud	625	625	100,00%	623	2	0,32%
Città di Aprilia	580	400	68,97%	398	2	0,50%
Belcolle Viterbo	1234	1234	100,00%	930	304	24,64%
San Camillo de Lellis	526	418	79,47%	413	5	1,20%
Fabrizio Spaziani FR	1352	1352	100,00%	1067	285	21,08%
Santissimia Trinità FR	910	491	53,96%	486	5	1,02%
Santa Scolastica	695	703	101,15%	569	134	19,06%
Total	44,805	41,510	92.65%	39,217	2891	7.37%

First stage screening in the birth centers of the Lazio region in 2016

Incomplete data reporting from Level II facilities did not allow a consistent analysis of second stage screening. Data were received only from four centers (36.4%), in which second stage screening was performed on 1175 children; among these children, 49 (4.2%) had own or maternal specific risk factors. One hundred-twenty-five children (10.6%) failed second stage screening and were referred to Level III centers for clinical diagnosis of hearing loss.

Third stage evaluation was performed on 365 children in the Level III centers of the Lazio region. They included children that failed second stage testing and children that were referred by other hospitals for risk factors. Data are summarized in Table 4.

- *Policlinico Umberto I*: 44 children were referred for clinical hearing evaluation; 33 were children that did not pass second stage screening (21 in the same center, 11 in a different hospital), 12 were referred for risk factors. Two children dropped out and could not be tested. Of tested children, 18 had normal clinical ABR in both ears and were considered having normal hearing; 24 were diagnosed with clinical hearing loss (13 unilateral, 11 bilateral), and they included four children with risk factors. Children with bilateral hearing impairment had profound hearing loss in two cases, severe in three cases, and moderate in six cases; children with unilateral hearing impairment had profound hearing loss in three cases, severe in two cases, moderate in five cases, and mild hearing loss in three cases.
- *Policlinico Gemelli*: 114 children were referred to this center for clinical hearing evaluation; 39 failing second stage screening (32 from same center, 7 from a different center) and 75 for risk factors. Sixty-six children dropped out and could not be tested. Forty-eight children underwent clinical ABR; 31 had normal hearing and 17 were diagnosed with clinical hearing loss (nine unilateral, eight bilateral). All children with bilateral hearing impairment had a moderate hearing loss in both ears (one had moderate hearing loss in one ear and mild in the other); children with unilateral hearing impairment had a profound hearing loss in two cases, severe in one case, mild in six cases.
- *Ospedale Pediatrico Bambino Gesù*: 207 children were tested with clinical ABR. Of these, 29 were diagnosed with unilateral (6/29) or bilateral (23/29) hearing loss. Children with bilateral hearing impairment had profound hearing loss in 11 cases, severe in four cases, and moderate in seven cases; children with unilateral hearing impairment had profound hearing loss in one case, severe hearing loss in two cases, and moderate in three cases.

**Table 4:** Third stage screening results

	Policlinico Umberto I	Policlinico Gemelli	Bambino Gesù
Included (RF)	44 (12)	114 (75)	207
Tested (%)	42 (95.4)	48 (42.1)	207 (100)
Normal Hearing (%)	18 (42.9)	31 (64.6)	178 (86)
Hearing Loss (%) [RF]	24 (57.1) [4]	17 (35.4)	29 (14)
Unilateral (%)	13 (54.2)	9 (52.9)	6 (20.7)
Mild (%)	3 (23.1)	6 (66.7)	-- (−)
Moderate (%)	5 (38.5)	-- (−)	3 (50)
Severe (%)	2 (15.4)	1 (11.1)	2 (30)
Profound (%)	3 (23.1)	2 (22.2)	1 (20)
Bilateral (%)	11 (45.8)	8 (47.1)	23 (79.3)
Mild (%)	-- (−)	-- (−)	-- (−)
Moderate (%)	6 (54.5)	8 (100)	7 (30.4)
Severe (%)	3 (27.3)	-- (−)	4 (17.4)
Profound (%)	2 (18.2)	-- (−)	11 (47.8)

Third stage screening performed in Level III centers of the Lazio region in 2016. *RF*: Risk Factors

The total number of children diagnosed with unilateral or bilateral hearing impairment during 2016 by Level III centers in the Lazio region was 70, with a prevalence of 1.6/1000. Children with a diagnosis of unilateral hearing loss were 28 (0.62/1000), while children with bilateral hearing impairment were 42 (0.93/1000). Incomplete data reporting does not allow calculating specific data for well born babies and babies with risk factor.

## Discussion

This is the first study that analyses the results of the UNHS in the Lazio region. Previous studies in other Italian regions include Liguria [18], Umbria [19], Tuscany [20], Campania [21] and Sicily [22], while other reports focused on specific cities or hospitals, such as the Ferrara [23], Pisa [24], Parma [25], Milan [26] and Siena experiences [27]. Our study shows a coverage rate in the Lazio region in 2016 of 93.3%, which is close to the recommendations by the JCIH guidelines that require a minimum coverage rate for the newborn population of 95% and a percentage of false positive < 3% [14]. The coverage rate of the Lazio region is also in line with national averages, that have consistently increased over the past years. In the past, a study from Bubbico et al. [28] investigated the evolution of the coverage rate of UNHS in several Italian regions from 2003 to 2011, showing a progressive increase of coverage that reached in 2011 a national average of 78.3%; coverage exceeded 95% in 12/20 regions especially in the North West and North East regions, while some areas such as the main islands still showed a limited diffusion of hearing screening programs. In 2017 the Italian Ministry of Health introduced UNHS among the ELA; this will allow a further increase of the coverage rate in the upcoming years.

UNHS in the Lazio region allowed identification of 70 children diagnosed with unilateral (40%) or bilateral (60%) hearing loss during 2016, with a prevalence of 1.6/1000. This result is close to estimates in the United States that range from 1 to 3 per 1000 newborns [1] and to that of other Italian studies [18, 20, 24, 25, 27]. A study on prevalence of prelingual deafness in Italy published in 2007 showed 40,887 cases of prelingual sensorineural hearing impairment ≥60 dB, with a prevalence in the Italian population of 0.72 per 1000 inhabitants [29]. These results could therefore confirm the validity of the screening organizational model developed in the Lazio region.

A significant number of children with a clinical diagnosis of hearing impairment had unilateral hearing loss, a condition that is often underestimated and untreated, in part because of lack of awareness of possible consequences [30–33]. Usually, children with unilateral hearing loss enter a protocol of subsequent follow-up to monitor a possible evolution into bilateral forms. Also, screening may miss mild permanent hearing loss misdiagnosing it with a transitory conductive form. Due to the high percentage of unilateral hearing loss, it is therefore important to take this condition into account when evaluating and, especially, predisposing an intervention plan.

Our study highlighted several difficulties in the current procedures and methodologies of the UNHS program performed in the Lazio region that may also apply to other realities. One of the main issues is the incomplete data collection from Level II facilities due the absence of a functioning reporting network between these facilities and Level I and III structures, further worsened by the lack of coordination from central regional offices. Our analysis is based on data from about 35% of Level II centers; this did not allow a consistent analysis of second stage screening with potential serious consequences not only on UNHS monitoring, but also on the effectiveness of the program with increased number of lost to follow-up (LFU) children and delayed diagnosis of hearing loss. Furthermore, the lack of data sharing from Level II facilities does not allow differentiating between well born children with a “refer” response at first stage and children with risk factors that are sent directly to second stage screening. Data from Level I facilities are transmitted and tracked by the monitoring office at the Lazio region allowing correct reporting of coverage. It would be auspicial to also extend such monitoring activity to Level II and Level III facilities, as already in place in some other Italian regions [18, 19].

There are some additional important data that are missing from this study. The first is the number of babies with risk factors. A study from Ghirri et al. [24] reported a prevalence of 6.1% in the Pisa hospital; Molini [19] reported a prevalence of 3.8% in Umbria. Data from the literature describe a prevalence between 3% and 5% of neonatal risk factors for hearing loss in the general population. In the Lazio region, the number of children with neonatal or maternal risk factors was not tracked at a central level, and therefore this data was not included in the present study. Secondarily, but not less important, age of patients included in third stage evaluation is unknown. Data for this stage were received directly from Level III facilities and were collected using different methodologies. Taken into account that all patients in Level III facilities followed the entire screening protocol, it could be assumed that diagnoses were performed within the first year of life. However, missing age data did not allow us to exactly evaluate the mean age of diagnosis. Moreover, no information about the beginning of audiological treatment was received.

Data about auditory neuropathy spectrum disorders (ANSD) were not available. In children with ANSD, the integrity of outer hair cells results in a “pass” response at TEOAE and therefore auditory dysfunction may remain undiagnosed at first stage evaluation [34–36]. To reduce the risk of undiagnosed ANSD, it is recommended to initiate auditory monitoring in babies with risk factors and instruct families of children who pass first stage to perform a further audiological evaluation if they notice abnormalities of auditory behavior in their children. Data from ANSD have not been collected in the present study; however, prevalence and natural history of ANSD is still largely unknown and possible revision of current screening protocols may be warranted as more information on these disorders become available [27].

LFU babies (newborns who fail the initial testing and are lost during follow-up) represent a common and alarming problem in UNHS [37–39]. In our study, data from Level III facilities showed an LFU rate < 5% for 2 centers, and a very high rate for the third center (Policlinico Gemelli). The data from the third center could be explained by regional or national mobility of these patients and may be biased by the unexpectedly very high number of babies with risk factors reported by that center. Instead, data about LFU patients between first and second and between second and third stages are not available. The collection of this data is necessary and important to monitor and actuate intervention strategies aimed to limit the number of LFU patients, such as targeted training to the personnel involved in screening procedures and appropriate education about UNHS and the risk factors of undiagnosed hearing impairment to pediatricians, neonatologists and gynecologists, and families. Better communication between centers and central regional offices is of utmost importance to limit LFU patients and increase effectiveness of UNHS.

Last, a noteworthy problem of UNHS that was encountered in our analysis is the heterogeneity between Level I centers in performing an A-TEOAE retest on refer children before addressing them to Level II centers. The utility of this retest has been demonstrated in several studies, allowing a drastic reduction of false positives and, therefore, of the workload for Level II and III facilities [24, 40]. However, in the Lazio region not all facilities perform such retest; this should be corrected and could explain the high variability of refer patients between Level I centers that ranged between 0% of some birth centers to over 20% of others (see Table 3).

## Conclusions

A correct and early diagnosis of hearing loss is mandatory to prevent its linguistic, developmental and educational consequences; the spread of UNHS programs is the optimal solution to reach this goal. The implementation of a UNHS program at a regional level requires consistent organizational efforts and is characterized by interconnected processes, activities and reporting. The analysis of 2016 UNHS in the Lazio region – although preliminary - allowed identification of several strengths and weaknesses of the program. The strengths included a correct spread and monitoring of UNHS among Level I facilities, with an adequate coverage rate, and the proper execution of audiological monitoring and diagnosis among Level III facilities. Weakness, instead, mainly consisted in lack of an efficient and automated central process for collecting, monitoring and reporting of data and information. Such lack may affect the overall effectiveness of the screening program; in fact, although single participants involved in the UNHS program appeared to be fully responsible for their activity, the central coordination by the Lazio regional offices only included the first stage of screening and is currently not extended to Level II and III facilities. It would be auspicial to increase effectiveness of the screening program and to favor a constant bidirectional flow of data between centers, stages and central monitoring offices.

## Abbreviations

ABR: Auditory Brainstem Responses
ANSD: auditory neuropathy spectrum disorders
DPOAE: Distortion Product Otoacoustic Emissions
ELA: Levels of Assistance
JCIH: Joint Committee on Infant Hearing
LFU: lost to follow-up
NICU: neonatal intensive care units
TEOAE: Transient Evoked Otoacoustic Emissions
UNHS: Universal newborn hearing screening
